# Visualization of consensus genome structure without using a reference genome

**DOI:** 10.1186/s12864-017-3499-7

**Published:** 2017-03-14

**Authors:** Ipputa Tada, Yasuhiro Tanizawa, Masanori Arita

**Affiliations:** 1 Center for Information Biology, National Institute of Genetics, Mishima, Shizuoka 411-8540 Japan; 2 Department of Genetics, School of Life Science, SOKENDAI (The Graduate University for Advanced Studies), Mishima, Shizuoka 411-8540 Japan; 3 RIKEN Center for Sustainable Resource Science, Yokohama, 230-0045 Japan

**Keywords:** Comparative genomics, Circular visualization, *Lactobacillus casei*, *Helicobacter pylori*

## Abstract

**Background:**

Standard graphical tools for whole genome comparison require a reference genome. However, any reference is also subject to annotation biases and rearrangements, and may not serve as the standard except for those of extensively studied model species. To fully exploit the rapidly accumulating sequence data from the recent sequencing technologies, genome comparison without any reference has been anticipated.

**Results:**

We introduce a circular genome visualizer to compare complete genomes of closely related species. This tool visualizes the position of orthologous gene clusters rather than actual sequences or their features, thereby achieving the comparative view without using a single reference genome. The essential information is the matrix of orthologous gene clusters whose positions (not sequences) are color-coded in circular graphics. As a demonstration, comparison of 14 *Lactobacillus paracasei* strains and one *L. casei* strain revealed not only large-scale rearrangements but also genomic islands that are strain-specific. Comparison of 73 *Helicobacter pylori* strains confirmed their genetic consistency and also revealed the three general patterns of large-scale genome inversions.

**Conclusions:**

From the ample sequence information in the GenBank/ENA/DDBJ repository, we can reconstruct a genomic consensus for particular species. By visualizing multiple strains at a glance, we can identify conserved as well as strain-specific regions in multiply sequenced genomes. Positional consistency for orthologous genes provides information orthogonal to major sequence features such as the GC content or sequence similarity of marker genes. The positional comparison is therefore useful for identifying large-scale genome rearrangements or gene transfers.

**Electronic supplementary material:**

The online version of this article (doi:10.1186/s12864-017-3499-7) contains supplementary material, which is available to authorized users.

## Background

The taxonomic landscape of bacteria is drastically changing. Next-generation sequencers (NGS) rapidly reveal genomic differences between and within species, and the genome-wide similarity statistics such as the average nucleotide identity (ANI) are used to assist, or even replace, the traditional methods of bacterial taxonomy [1, 2].

By definition, every bacterial species is a collection of strains that are considered identical based on their phenotypic traits (culture growth) and DNA-DNA hybridization (DDH). The practical norm for specific identity has been greater than 70% DDH, but this assessment is notoriously cumbersome, onerous process. For example, the number of bacterial species described to date remains less than 5000, a significant underestimation in contrast to over 1 million eukaryotic species [3]. As a more efficient method, the sequence similarity of 16S rRNA has also been popular in the field of molecular genetics. Previously, 97% identity had been the standard norm to define the notion of species [4, 5]. This threshold was recently revised to 98.7–99.0% by the same author [6]. The method has greatly influenced and boosted biological studies, but its major drawback is a failure to identify genome-wide divergences such as gene gain/loss or horizontal transfers. Genome-wide statistics such as ANI is therefore expected to circumvent above difficulties and explores a new horizon with the new sequencing technologies.

It is noteworthy that all computational methods, including ANI, are based on binary comparison with good reasons. In microbiology, each bacterial (and archaeal) species must have a designated representative strain called the “type” (or alternatively reference strain), which is a living culture to define and maintain the taxonomy. To identify newly isolated species, in practice, taxonomists use a polyphasic approach against the type strains of known close relatives. The comparison includes verification of overall similarity based on multiple characteristics including phenotypic and phylogenetic traits. For this purpose, type strains must be not only publicly available from stock centers but also have been under sequencing effort with priority to verify their published names and genomic diversity [7, 8].

In the era of NGS, the polyphasic comparison for taxonomic identification should include whole-genomic traits such as horizontal gene transfers or rearrangements. This view sheds a unique light to the definition of a species or ecotype, and subsequently its type strain. Finding whole-genomic traits is not straightforward; it is not immediately clear from the computational comparison of 16S rRNA sequences or ANI. For example, application of the ANI index has revealed that, even among strains showing >99% ANI, a genomic potential of bacteria in different ecological niches may vary drastically. On the other hand, current definition of species sometimes allows ANI values lower than the suggested lower limit of 95% [1].

Even more serious is a submission inconsistency in the public sequence repository (GenBank-ENA-DDBJ). Databases must rely on submitters for the correct taxonomic identification. A recent publication suggests, however, that as much as 18% of all prokaryotic species suffer from anomalies in the species definition [9]. Incorrect use of scientific names is also prevalent in scientific papers. Researchers, especially bioinformaticians, do not care about taxonomic accuracy; they only copy and paste scientific names from databases or previous literature. In this situation, it would become extremely difficult especially for beginners to notice whether the genomes they manipulate are correctly annotated and deposited. One solution is a visualization tool that can output taxonomic anomaly at a glance to help resolving such issues.

In this report, we introduce a visualization method for genome sequences of closely related strains. Several visualizers have been proposed to date [10–13], but our approach is unique in that we do not presuppose binary comparison between genomes. Comparison against a single reference implicitly assumes the perfection of the reference data. Practically, however, it is not guaranteed at least for two reasons. First, the reference genome is also subject to rearrangements or gene loss/gain as easily as any other strain within the species. Second, annotation is always subject to human errors. To become free from rearrangements or annotation errors that may occur in any strain, we need a visualizer that can detect the genomic consensus out of available, multiple strains that belong presumably to the same species.

To achieve the robustness we require for visualizing species consensus, our method uses the relative position (in degree) of homologous genes within each genome. This intuitive strategy functions well for closely related strains. We demonstrate its effectiveness by using two exemplary bacterial species: *Lactobacillus (para)casei* and *Helicobacter pylori.* The former is a well-known case of scientific taxonomic controversy [14]. We compare the type strain of *L. casei* (ATCC 393) with 14 *paracasei* strains, among which eight strains are still referred to as *casei* strains in databases and many scientific papers (they are indistinguishable from rRNA sequences and other assays). Although their genomic structures are similar, we show their difference in gene locations, which becomes evident in our circular visualization. The other example is *H. pylori*, an obligatory pathogen from human stomach. This species is known to keep the same gene contents with substantial nucleotide changes as a pathogen in a highly restricted ecological niche [15]. We delineate its frequent genome inversions and rearrangements with the circular graphics. Most of all, we exemplify that our tool can detect not only genome rearrangements but also annotation biases, such as the rotated shifts and possible mis-assemblies. Such anomalies are difficult to locate without graphical presentation at the time of data submission or inspection.

## Methods

### Genome sequences

Genome sequences for 15 *Lactobacillus* strains (*paracasei* KL1, *paracasei* N1115, *casei* subsp. casei ATCC 393, *paracasei* CAUH35, *paracasei* subsp. paracasei JCM 8130, *casei* LOCK919, *casei* 12A, *casei* str. Zhang, *paracasei* subsp. paracasei 8700:2, *paracasei* ATCC 334, *casei* W56, *paracasei* L9, *casei* BL23, *casei* BD-II, and *casei* LC2W) were obtained from the GenBank/ENA/DDBJ repository. Although 8 species were labeled as ca*sei* by their submitters, only ATCC 393 is the true *casei* strain and all others are *paracasei* in the current standard definition. For justification with the ANI matrix of 15 strains, readers are referred to (Additional file 1: Table S1).

Total 73 strains of *H. pylori* were also obtained from the GenBank/ENA/DDBJ repository. They were annotated as 2017, 2018, 26695, 26695, 26695–1, 26695–1, 26695-1CH, 26695-1CL, 26695-1MET, 29CaP, 35A, 52, 7C, 83, 908, Aklavik117, Aklavik86, B38, B8, BM012A, BM012B, BM012S, BM013A, BM013B, Cuz20, ELS37, F16, F30, F32, F57, G27, Gambia94/24, HUP-B14, Hp238, India7, J166, J99, Lithuania75, ML1, ML3, NY40, OK113, OK310, P12, PeCan18, PeCan4, Puno120, Puno135, Rif1, Rif2, SJM180, Santal49, Sat464, Shi112, Shi169, Shi417, SouthAfrica20, SouthAfrica7, UM032, UM037, UM066, UM298, UM299, XZ274, oki102, oki112, oki128, oki154, oki422, oki673, oki828, oki898, and v225d. For strain details and their ANI matrix, see (Additional file 2: Table S2). The strain 26695 was twice registered by two different institutions (TIGR and RIPCM) and the strain 26695–1 was twice registered by Oita university.

### Choice of ANI index

There are several ways to compute the ANI value [9]. We calculated ANI by counting the number of identities across the gapped pairwise alignment between two genomes by customizing the open-source Python script contributed by Leighton Pritchard (James Hutton Institute) at the GitHub source-code repository [16]. The method does not compute the fraction of each genome contributing to the alignment, but was chosen for efficiency and transparency.

### Finding gene clusters

Protein BLAST (version 2.2.29+, e-value < 1e-5) was performed for the set of genomes and result tables were combined into orthologous gene clusters by the bidirectional best-hit (BBH) criterion. The maximum size of each gene cluster was therefore the number of genomes used: 15 for *Lactobacillus* and 73 for *Helicobacter*. Genes in the clusters were assigned their coding loci in degree angles (0–359 integers) starting from the angle 0 position in each genome. For each gene cluster, its average, median, and standard deviation of member-gene angles were computed. When the standard deviation of gene angles was equal or lower than five (within the range of 360), the average value was used as the position angle of the gene cluster. When it was more than five, the median value was used as the cluster angle, because the average value might not correspond to the position of any member gene. The set of all cluster positions was regarded as the consensus genomic structure.

### Genome alignment and visualization

When all gene clusters obtain their degree positions (the consensus genome), we can compute a distance for each genome from the consensus by calculating the sum of deviations of all orthologous genes in the genome from the consensus. All genomes were sorted by their deviation in the descending order, and circularly visualized from the outermost ring (number 1) inward. The outermost ring was therefore most distant from the consensus. The standard customizable software Circos was used for visualization [10].

After creating the consensus genome, any genome can be aligned to the consensus by minimizing the sum of degree differences of all gene clusters. The alignment inevitably becomes an iterative process because the rotation of any genome will change all positions of orthologous clusters. Although most genomes were similarly annotated, some genomes required such alignment by rotating the whole sequence. Others also required flipping to align, i.e., using their reverse complements. See the main text for details.

### Multidimensional scaling (MDS) and x-means clustering

Multidimensional scaling (MDS) plot and heatmap were created by the R package (version 3.2.4) with reshape2 and ggplot2 libraries [17]. MDS plot was performed with the deviation of core genes that were coded in all strains investigated. The clustering with the x-means algorithm was written in R scripts [18].

## Results

### Circular visualization of consensus genome

For visualization of closely related genomes, we used their orthologous gene clusters detected through the bidirectional best-hit (BBH) by Protein-BLAST (see Methods). Genes that were not included in BBH were not considered in this work. We call the set of orthologous clusters with their genomic positions as the *consensus genome*. Once the consensus is determined, its visualization can be adjusted by user-selected values, such as the minimum number of genes in each cluster (from strain-specific genes to core genes) or the positional deviation of each cluster to show genome rearrangements and transposable elements. In this analysis, we shall focus on large-scale genomic rearrangements.

**Fig. 1:**
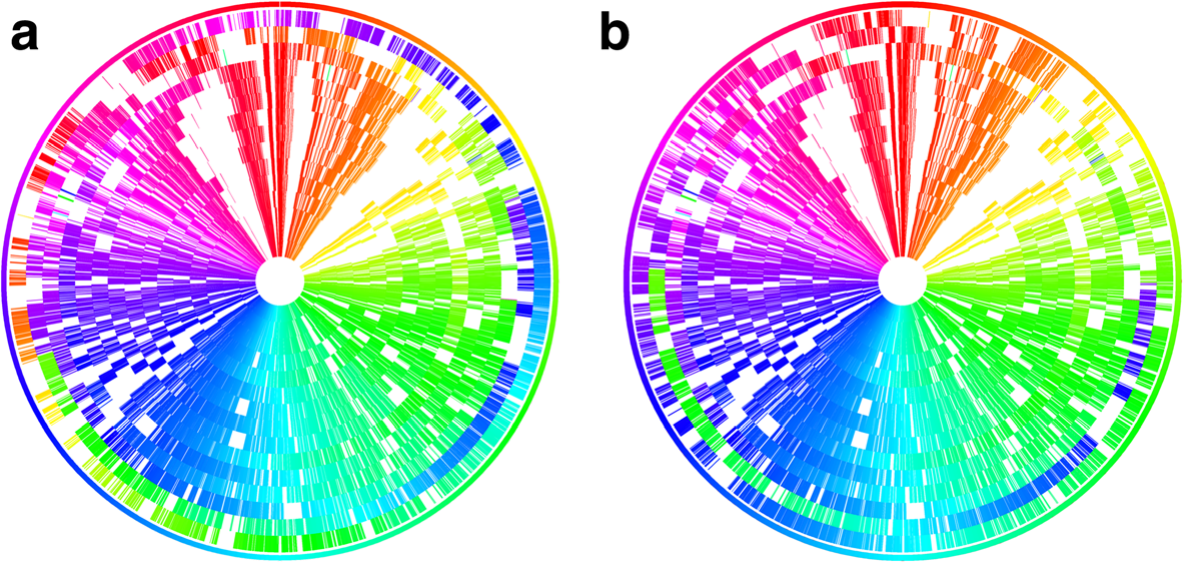
Circular view of *Lactobacillus paracasei* and *L. casei* (3^rd^ ring from the outmost). **a** Core genes shared by all strains without alignment. Two outmost rings are apparently unaligned. **b** Core genes after alignment. Two outmost rings fitted with the others and the second outmost strain (*paracasei* N1115) showed a large genome inversion

To highlight rearrangements, genes are color-coded by the genomic position (in degree) of the cluster they belong to. When all genes in the same orthologous group are coded at the same genomic locus (within 5° range from the average by default), the same color appear at the same position in circular views. If a small subgroup of the orthologous genes are relocated to a different locus, the color of the relocated small group will become different from their neighboring genes because the color comes from their larger sibling group in a different position (Fig. 1). To realize such coloring, gene color is determined by the majority rule, i.e., the color is chosen by the degree position in which most number of genes reside in each cluster. The software program was written in Bash, Perl, Python, and R. The program source codes are available on request from the authors.

### Genome-scale comparison of *Lactobacillus paracasei* and *L. casei*

In Fig. 1, we show 1525 core genes (genes shared by all investigated strains) of 14 *Lactobacillus paracasei* and 1 *Lactobacillus casei* (the 3^rd^ outmost ring). Their genome sequence ranged 2.77–3.11 Mb in size, with a GC content of 46–47%. The average genome size of 2.97 Mb was close to the size of *L. casei* strain (2.92 Mb) and all genome sizes were similar. The average number of genes was 2901 (2763 proteins), and the pan-genome size was 4187. We did not use the standard Markov clustering method for finding orthologues, because our approach required strictly one-to-one orthology among genes. The number of core and accessory genes by BBH was similar to a previous report of comparative study that used Markov clustering [19]. The slightly smaller number of core genes was due to the inclusion of the *L. casei* strain.

When all the complete genomes were visualized as registered in the sequence repository (Fig. 1a), we could immediately see the rotated shift for the two outmost rings (*paracasei* KL1 and N1115). When they were aligned to the consensus (see Methods; Fig. 1b), the large genome inversion for *paracasei* N1115 was evident, spanning half of its genome. Uncolored positions roughly corresponded to genomic islands, where gene sequences are species-specific (this is the nomenclature in the *Lactobacillus* community) [20]. The direction of 11 o’clock is populated with many metabolic genes in *Lactobacilli* and therefore not shared (uncolored) [21]. The direction of 1 and 2 o’clock is also populated with carbohydrate utilization genes, e.g. phosphoenolpyruvate-carbohydrate phosphotransferase (PTS)-type transporter systems or glycosyl hydrolases, and therefore uncolored [20]. The benefit of our graphics is that such trends are visible at a glance.

**Fig. 2:**
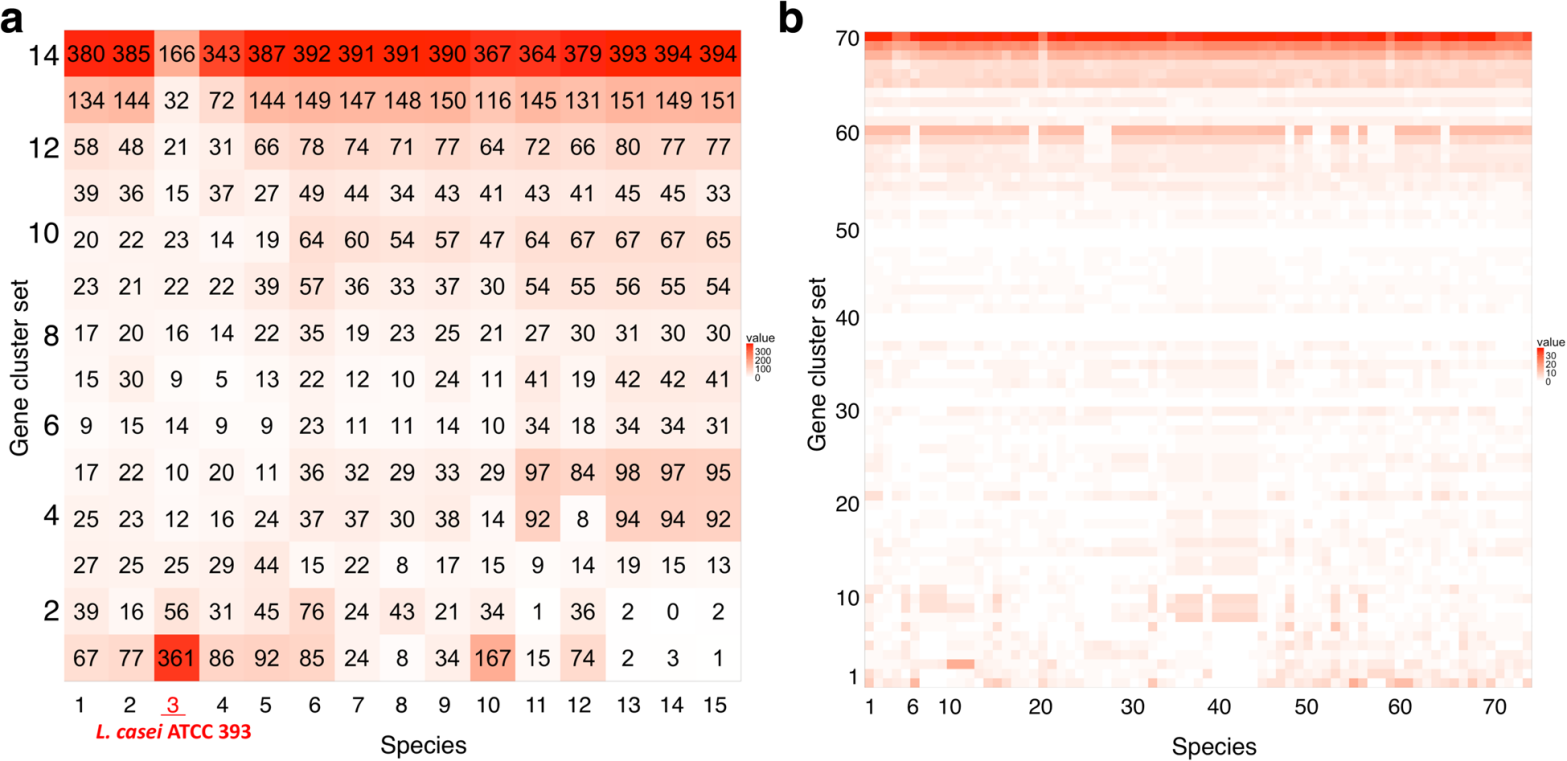
Heat map of shared genes in *Lactobacillus* **a** and *Helicobacter* **b**. High resolution data with all strain names and the corresponding ANI calculations are available as (Additional file 3: Figure S1 and Additional files 4: Figure S2)

The difference between *L. casei* and *L. paracasei* is also identifiable although their rRNA sequences are extremely similar. When the genomic inversion of *paracasei* N1115 (2^nd^ outmost ring) in Fig. 1b is flipped (figure not shown), the *casei* ATCC 393 strain (3^rd^ outmost ring) has conspicuous changes such as the genomic shift of the 3 o’clock region into 2 o’clock (green zone entering yellow), and the overall color shift between 11 and 2 o’clock. The difference in gene contents was also evident from the heat map of their orthologous clusters (Fig. 2a) and the ANI calculation (Additional file 3). Only *L. casei* ATCC 393 strain contained as many as 361 singletons (the bottom row of Fig. 2a), and the second most singletons was 167 for *paracasei* ATCC 334. The number of common orthologues was also the least for *casei* ATCC 393 (166 genes only; the topmost row). All others shared as many as >340 genes. In summary, our method effectively visualizes large-scale changes in multiple genomes.

### Justification of consensus formation

The reference-less method critically depends on the formation of consensus structure, i.e., the average position of orthologous genes. To check the distribution of gene positions quantitatively, we calculated the positional shift (in degrees) of orthologous genes in each of 15 *Lactobacillus (para) casei* strains (Table 1). The number of genes deviating from the consensus position showed a clear difference. In *casei*, most genes were shifted for 11–15° from the consensus while the shift were within 5° for all the other *paracasei* strains. This genome-scale difference was effectively visualized in our method. As the second example, we tried a larger set of genomes.

**Table 1:** The number of genes that are shifted from the consensus position. The first strain (ATCC393) is *Lactobacillus casei* and the rest are all *Lb. paracasei.* Only ATCC393 showed a different distribution and its standard deviation is the largest

Degree	ATCC393(*casei*)	KL1	CAUH35	N1115^a^	JCM_8130	LOCK919	W56	12A	Zhang	BL23	L9	BD-II	LC2W	8700	ATCC334	Average
0–5	537	1737	1547	1789	1778	1952	1787	2004	2311	1823	2287	1916	1794	2351	2393	1867
6–10	492	559	443	536	589	577	706	383	86	725	167	618	731	65	31	447
11–15	930	6	171	16	16	33	20	12	4	21	21	24	26	18	14	89
16–20	132	8	9	6	8	12	12	13	1	12	4	12	12	8	15	18
21–25	79	4	1	5	4	5	19	1	0	19	10	17	20	0	3	12
26–30	3	9	0	3	11	13	3	2	0	3	3	3	1	2	4	4
31–90	107	68	63	48	45	47	52	12	23	35	32	33	18	34	35	43
91–180	27	4	20	6	16	4	3	4	8	8	9	3	2	2	2	8
Median degree	11	4	3	2	3	2	3	3	3	3	2	3	2	2	1	3
Average deviation	19.4	9.9	8.6	6.8	6.1	5.8	4.4	4.4	4.4	4.3	4.2	4.2	4.1	3.6	3.4	6
Standard deviation	52.9	42.5	31.3	30.8	25.2	23.6	18.0	20.0	21.5	17.6	22.2	17.5	17.6	19.1	21.5	25

^a^A large inversion of N1115 strain was manually modified

### Genome-scale comparison of *Helicobacter pylori*

**Fig. 3:**
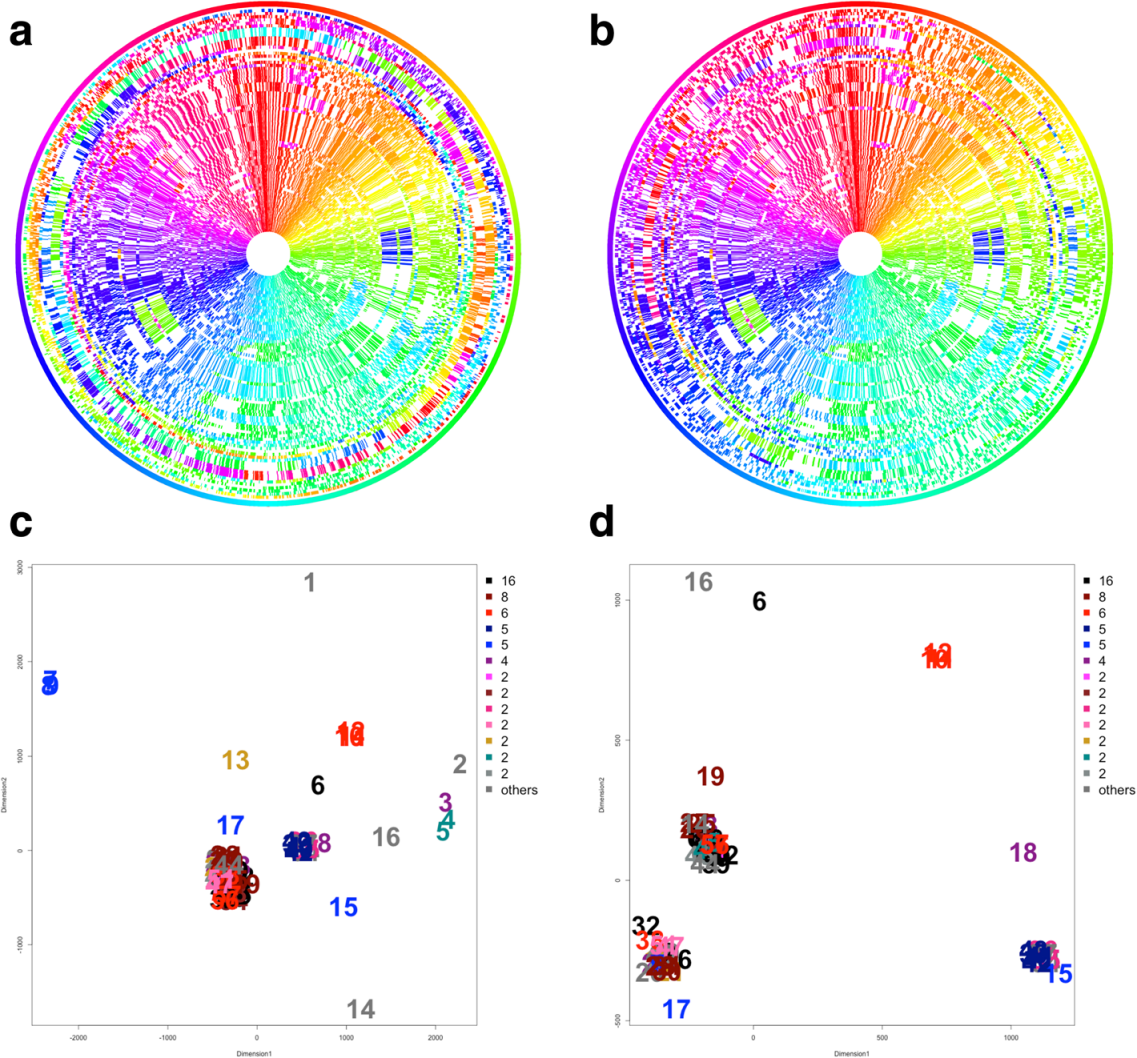
Circular view of all 73 *Helicobacter pylori* strains and their MDS plot based on the correlation distance. **a** Before rotation of the outmost 21 genomes. **b** After rotation of the outmost 21 genomes. **c** MDS plot of before the rotation. Colors indicate research groups. **d** MDS plot of after the rotation

In Figs. 2b and 3, we show a heat map and two circular views of 73 *H. pylori* strains*.* The genome size ranged from 1.49 – 1.71 Mb with the average of 1.63 Mb. The average number of genes was 1571 (1454 proteins), and the pan-genome size was 1871. This species is known for its extremely consistent gene content regardless of extensive nucleotide changes due to its niche habitat. Such features are readily visible in these figures. In contrast to *Lactobacillus* (Fig. 1), whose habitat is diverse including dairy, plant and gut isolates, *Helicobacter* exhibited much fewer genomic islands (colorless area) despite their frequent genomic rearrangements (color changes). In the heatmap, each *pylori* strain contained much fewer strain-specific genes than did *Lactobacillus*.

Higher ratio of core genes was also implied by the number of genomes compared. In *Lactobacillus*, the number of core genes decreased more rapidly as the number of compared genomes increased. The number of core genes (1525) was almost half of the average (2909) when the number of genomes was 15. In *Helicobacter,* on the other hand, half genes were still shared by as many as 73 strains (744 genes among the average 1571). This strong reverse-correlation between the number of core genes (genetic consistency) and the habitual diversity was also supported by the function of strain-specific genes. In genomic islands of *Lactobacillus*, sugar utilization genes vary depending on their isolated source or environment [19]. *Helicobacter* also lacks many genes for sugar metabolism and the genes in its plasticity region (this is the nomenclature for strain-specific regions in the *pylori* community). This region is known to involve with its pathogenicity [22].

### Three types of genome rearrangements in *H. pylori*

**Fig. 4:**
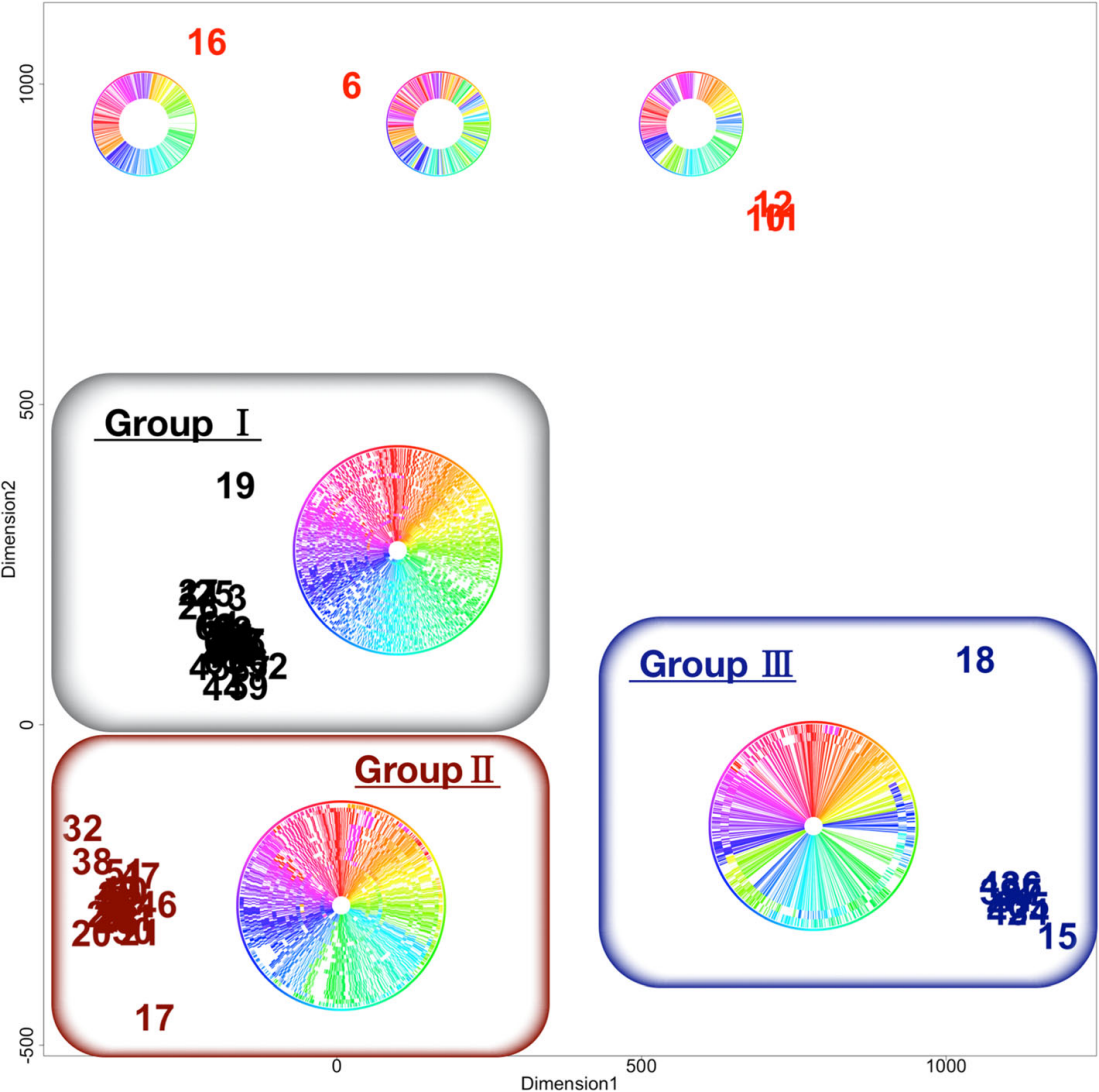
Circular view of three *pylori* groups in the MDS plot

Interesting feature in *Helicobacter* was that genomic rearrangements were roughly clustered into three groups. When the genomes were compared as registered in the data repository, many genomes were apparently unaligned (Fig. 3a). Application of MDS analysis showed that genomes with early numbers (1 to 21), corresponding to outermost rings, were distant from the remaining groups (Fig. 3c). We therefore rotated genomes of the 21 strains and inverted 9 strains to obtain the circular view of Fig. 3b. After alignment, most were clustered into three groups (Fig. 3d). In Fig. 4, we show genome rearrangements in each group.

Group I contained the largest number of strains from all continents (34 genomes), and was closest to the consensus genome by the majority rule. This group included famous strains such as the ulcerogenic J99 from North America (Nr. 14). Group II contained 23 strains, all of which included three genomic inversions in comparison with Group I: one nested inversion between 11 and 1 o’clock direction and the other, 4 and 7 o’clock direction (see color changes in Fig. 4). This group included many strains from East Asia but also included strains from North/South America. The plasticity region was visible in 7–8 o’clock direction (colorless region), and this structure was concordant with previous reports [23]. The last Group III contained 11 genomes, among which seven were all 26695 strains. They contained one nested inversion: the outer inversion between 3 and 8 o’clock direction and the inner, between 5 and 7 o’clock direction, as was reported in an early comparative study between J99 and 26695 strains [24]. The three groups did not match with their geographical areas isolated, research groups, or phylogenetic lineages computed from specific genes [23, 25, 26].

In Fig. 4, five strains remained unclustered with the three major groups: Aklavik86, ELS37, BM012B, BM012A, and BM012S strains. These strains exhibited rare rearrangement patterns. Of all, the number of genome rearrangements in the Aklavik86 strain from Canadian Aboriginal community (Nr. 6) exceeded 140 [27], in contrast to the standard number of less than five This genome indeed showed many color changes, and its excessive difference may have originated in their 454 FLX Titanium DNA sequencing anomalies.

Three Australian strains, BM012B, BM012A, and BM012S (Nr. 10–12) were reported by the same research group [28], and contained two complicated inversions. One was nested between 3 and 7 o’clock direction and between 4 and 6 o’clock direction. The other one consisted of three inversions between 8 and 10, 10 and 12, and overall 8 and 12 o’clock direction. The last ELS37 strain from El Salvador (Nr. 16) showed a unique inversion between 8 and 1 o’clock direction.

## Discussion

### Advantages of genome visualization

Genomic inversions and their distribution within strains are not easily identified only from numeric analyses such as ANI or multilocus sequence typing (MLST), or from a set of binary comparison against a reference. Visualization is a powerful method when it is used in combination with such numeric analyses. Indeed, we could identify three major rearrangement groups in *Helicobacter* without using a reference genome. They do not represent any geographic region, and imply that the rearrangements occur non-randomly. That is, genomic structures of 68 *pylori* strains have converged to the three patterns by some unknown selection pressure. Detailed analysis on their rearrangement sites is ongoing and we look forward to finding the cause. One possibility is that the complete genomes were reconstructed by referencing already published genomes. If this were true, however, the rearrangement groups would correlate with research groups or publication order. Such relationship was not detected in our current analysis.

### Limitation and comparison with other approaches

The approach assumes the availability of multiple strains for the same species to delineate genomic rearrangements and possible annotation anomalies. Since the structure of a consensus genome is formed by the majority rule of orthologous genes, we require an ample number of strains enough for drawing statistical assessments, especially the test for normality. This necessitates at least six (preferably more than ten) genomes for comparison. Frequently sequenced microbes such as *Lactobacillus* or *Helicobacter* can satisfy this criterion but rarely sequenced organisms are not applicable.

## Conclusions

Many comparative studies were conducted for *Lactobacillus (para)casei* and *Helicobacter pylori*, but previous works mainly focused on sequence features, not their genomic locations. We developed a program to visualize genomic positions of orthologous gene clusters and detected major genome inversions and rearrangements. Of note, genome rearrangement patterns in *H. pylori* were grouped into three, and the strain composition was independent from the *pylori*’s migration from Africa with their human hosts. Through our graphical method, detection of large-scale changes as well as species-specific islands can be efficiently achieved. This information is orthogonal to the traditional sequence-based features, and contributes to the field of comparative genomics.

## Additional files

Additional file 1: **Figure S1.** ANI matrix of *Lactobacillus* (A) and *Helicobacter* (B). (PDF 1.16 mb)

Additional file 2: **Figure S2.** High resolution data of Figure 2: Heat map of shared genes in *Lactobacillus* (A) and *Helicobacter* (B). (PDF 1.08 mb)

Additional file 3: **Table S1.** 15 species *Lactobacillus (para)casei* genome information. (XLSX 40.0 kb)

Additional file 4: **Table S2.** 73 species Helicobacter pylori genome 469 information. (XLSX 42.5 kb)
