# Supplementary figures and images for: Visualization of consensus genome structure without using a reference genome
Source: BMC Genomics. 2017 Mar 14;18(Suppl 2):208. doi: 10.1186/s12864-017-3499-7 (PMC5374595; doi:10.1186/s12864-017-3499-7)

A

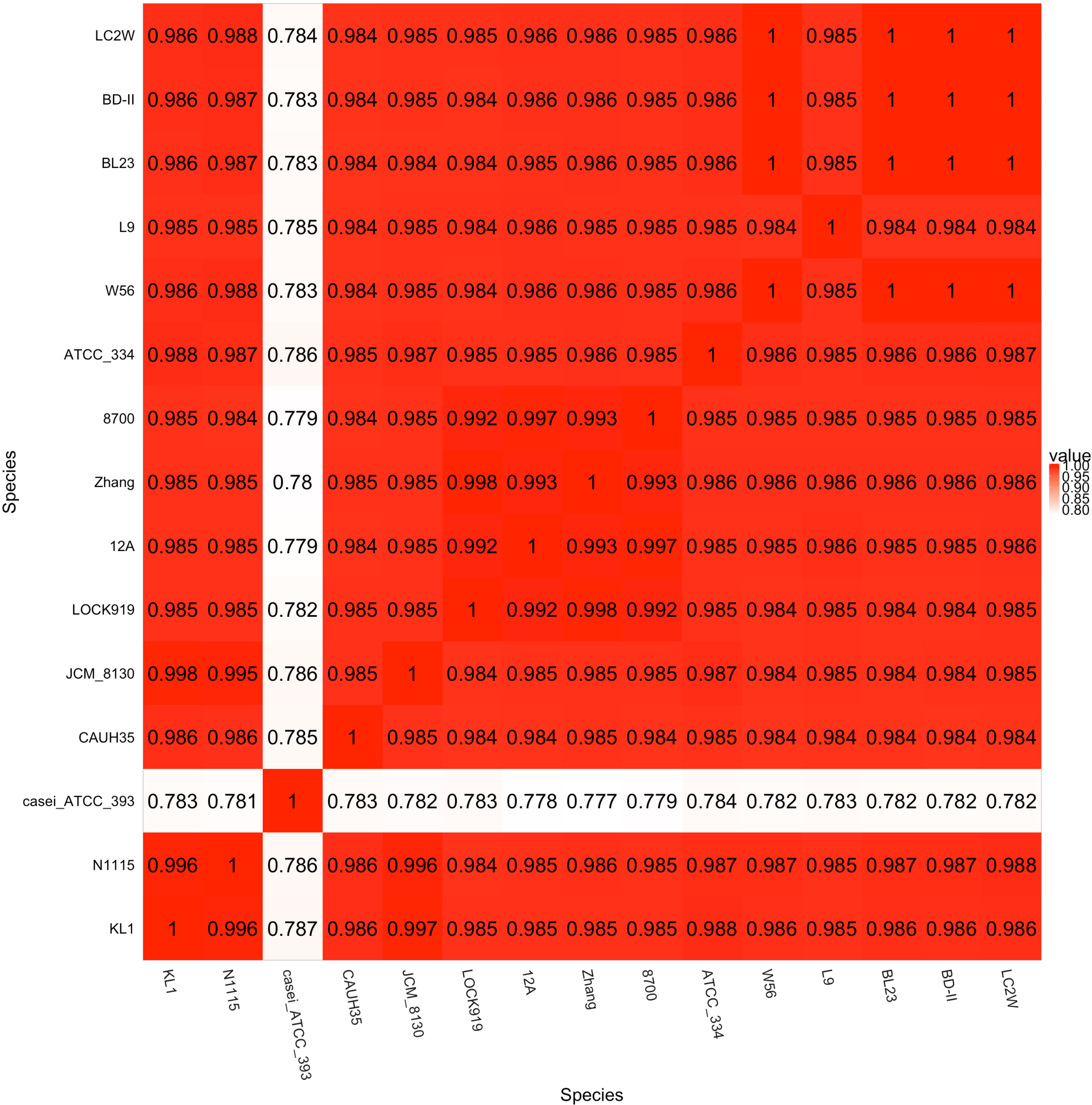

B

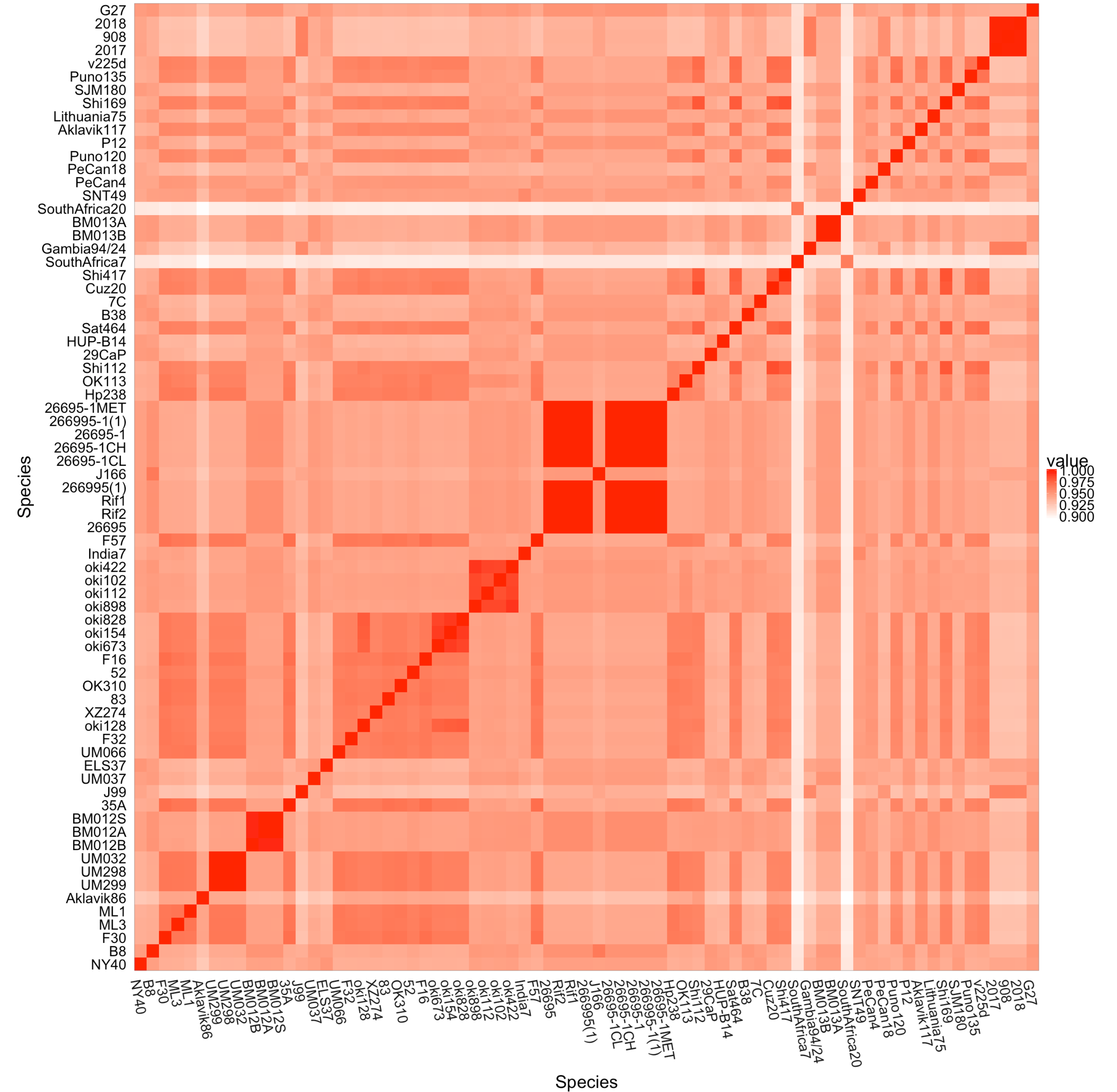

Supplement: Supplementary file 1 — Figure S1. ANI matrix of Lactobacillus (A) and Helicobacter (B). (PDF 1.16 mb) [file 12864_2017_3499_MOESM1_ESM.pdf]

**A**

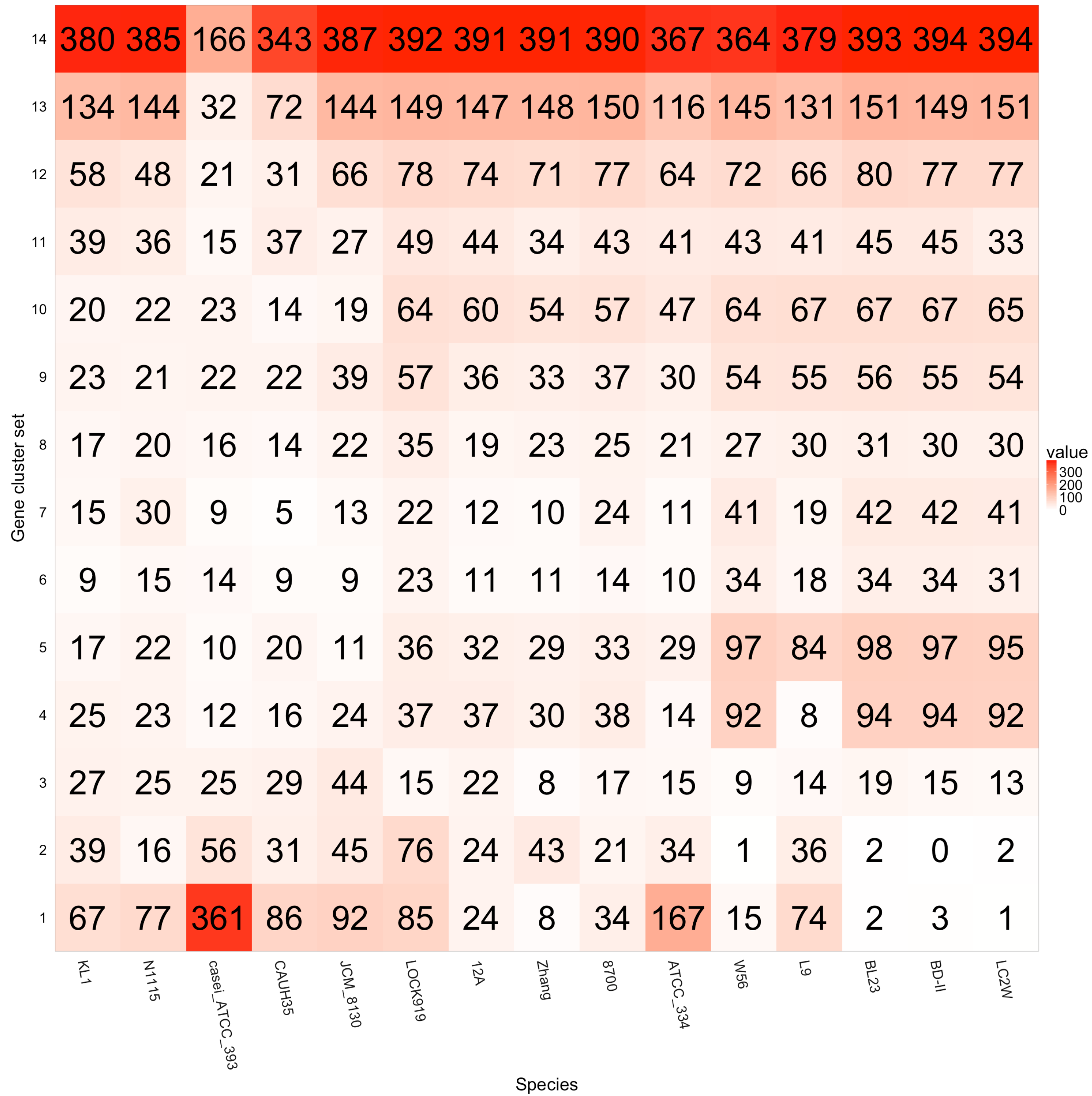

# B

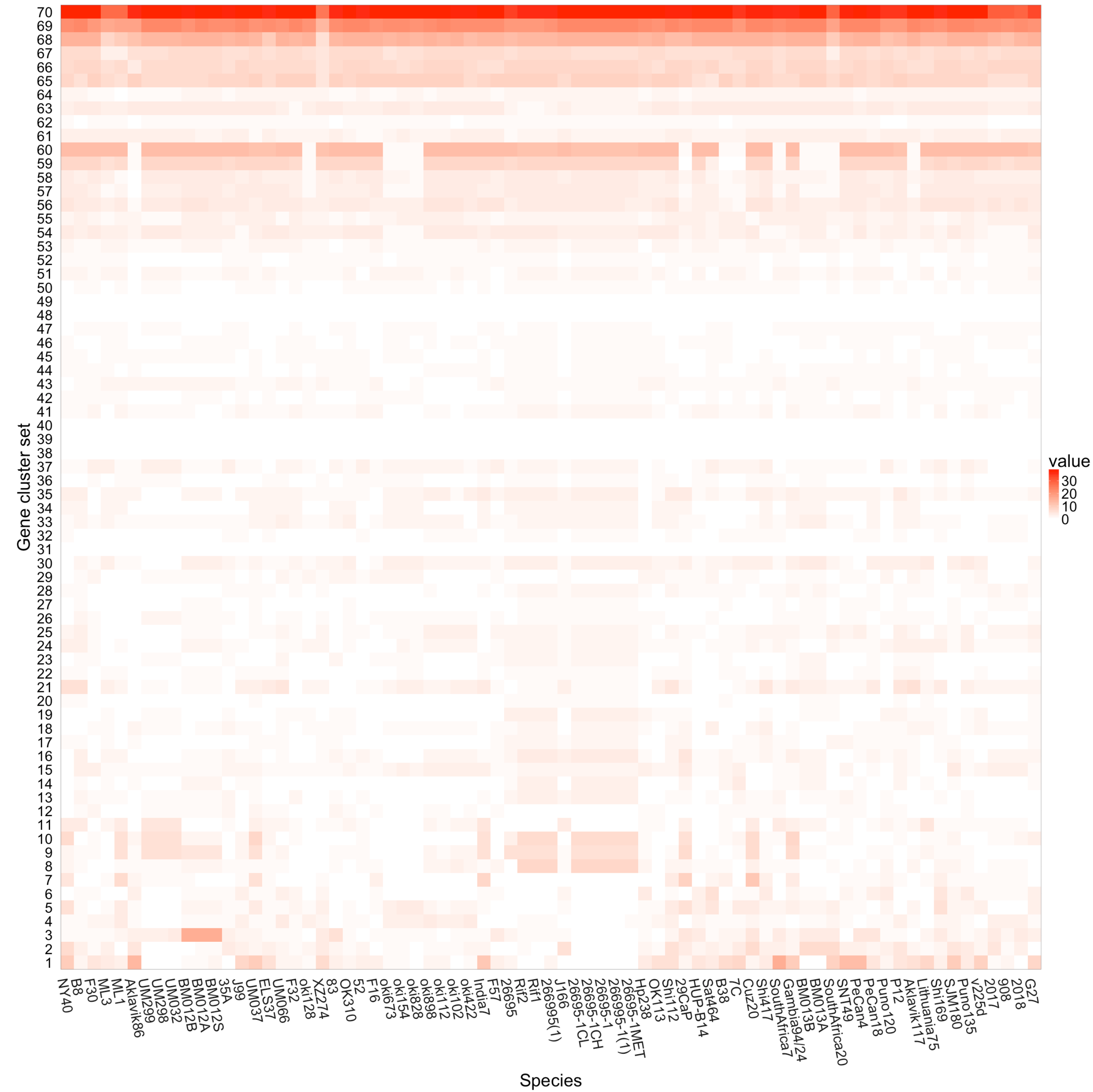

Supplement: Supplementary file 2 — Figure S2. High resolution data of Figure 2: Heat map of shared genes in Lactobacillus (A) and Helicobacter (B). (PDF 1.08 mb) [file 12864_2017_3499_MOESM2_ESM.pdf]
